# New Zealand’s School Dental Service over the Decades: Its Response to Social, Political, and Economic Influences, and the Effect on Oral Health Inequalities

**DOI:** 10.3389/fpubh.2017.00177

**Published:** 2017-07-31

**Authors:** Susan M. Moffat, Lyndie A. Foster Page, W. Murray Thomson

**Affiliations:** ^1^Faculty of Dentistry, Department of Oral Sciences, Sir John Walsh Research Institute, University of Otago, Dunedin, New Zealand

**Keywords:** New Zealand School Dental Service, Community Oral Health Service, dental caries, oral health inequalities, dental nurse, dental therapist, oral health therapist, dental therapy

## Abstract

New Zealand’s School Dental Service (SDS) was founded in 1921, partly as a response to the “appalling” state of children’s teeth, but also at a time when social policy became centered on children’s health and welfare. Referring to the Commission on Social Determinants of Health (CSDH) conceptual framework, this review reflects upon how SDS policy evolved in response to contemporary constraints, challenges, and opportunities and, in turn, affected oral health. Although the SDS played a crucial role in improving oral health for New Zealanders overall and, in particular, children, challenges in addressing oral health inequalities remain to this day.

Supported by New Zealand’s Welfare State policies, the SDS expanded over several decades. Economic depression, war, and the “baby boom” affected its growth to some extent but, by 1976, all primary-aged children and most preschoolers were under its care. Despite SDS care, and the introduction of water fluoridation in the 1950s, oral health surveys in the 1970s observed that New Zealand children had heavily-filled teeth, and that adults lost their teeth early. Changes to SDS preventive and restorative practices reduced the average number of fillings per child by the early 1980s, but statistics then revealed substantial inequalities in child oral health, with Ma¯ ori and Pacific Island children faring worse than other children.

In the 1990s, New Zealand underwent a series of major structural “reforms,” including changes to the health system and a degree of withdrawal of the Welfare State. As a result, children’s oral health deteriorated and inequalities not only persisted but also widened. By the beginning of the new millennium, reviews of the SDS noted that, as well as worsening oral health, equipment and facilities were run-down and the workforce was aging. In 2006, the New Zealand Government invested in a “reorientation” of the SDS to a Community Oral Health Service (COHS), focusing on prevention. Ten years on, initial evaluations of the COHS appear to be mostly positive, but oral health inequalities persevere. Innovative strategies at COHS level may improve oral health but inequalities will only be overcome by the implementation of policies that address the wider social determinants of health.

## Introduction

New Zealand’s School Dental Service (SDS) was established in the early twentieth century, at a time when social policy became centered on the health and welfare of children to better ensure the future success of the “race, nation and Empire.” As such, the SDS has been part of the structure of New Zealand’s oral health care system for close to 100 years and has had a formative influence on the lives of nearly all New Zealanders. Since its establishment in 1921, the SDS has had continued political support from successive Governments. This has focused on policies to improve the quality of care and interventions, access to care, and upskilling the workforce. Children’s oral health has improved considerably; however, oral health inequalities exist, with worse oral health outcomes experienced by Māori and Pacific Island children and adolescents, and children and adolescents living in areas of higher socioeconomic deprivation (1). This historical review focuses on key periods in the development of the SDS, as influenced by social, economic, and political factors, and critically examines the Service’s efforts to both improve oral health and, more recently, to reduce inequalities in oral health.

**Figure 1:**
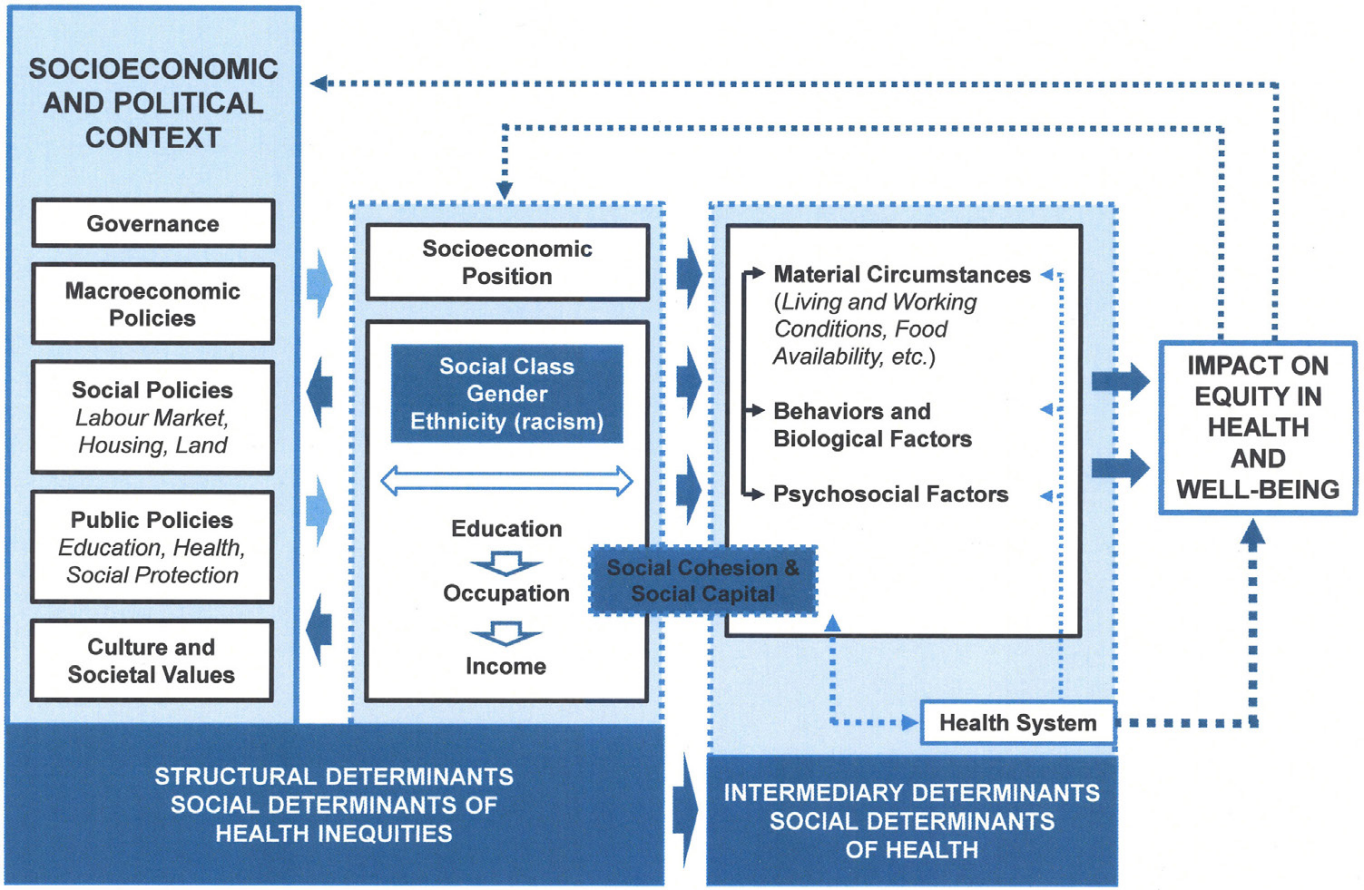
The Commission on Social Determinants of Health conceptual framework (2).

When examining the history of the SDS, it becomes clear that the socioeconomic-political context has had an impact on the service, and the inequities and inequalities in oral health that still exist. This broad term (socioeconomic-political) refers to the spectrum of factors in society that cannot be measured directly at the individual level. The Commission on Social Determinants of Health framework (Figure 1) shows how social, economic, and political mechanisms give rise to a set of socioeconomic positions, whereby populations are stratified according to income, education, occupation, gender, race/ethnicity, and other factors. These socioeconomic positions in turn shape specific determinants of health status (intermediary determinants) reflective of people’s place within social hierarchies. Based on their respective social status, individuals experience differences in exposure and vulnerability to health-compromising conditions (2).

The CSDH framework differs from many previous models in that it conceptualizes the health system itself as a social determinant of health (2). The SDS [now known as the Community Oral Health Service (COHS)] has been part of the New Zealand health system for almost a century and has developed over time as a result of the ever-changing social, political, and economic environment, thus impacting on oral health status in general and inequities in oral health. By utilizing the CSDH framework, we can explore the structural and social determinants that have impacted on the delivery of a service whose primary role was to improve the health and welfare of New Zealand children.

## Establishing a Dental Service for Children

At the first New Zealand Dental Association (NZDA) conference in 1905, F.W. Thompson, a well-known dentist, presented a paper entitled “the Teeth of our Children.” After examining the children at a Christchurch primary school, Thompson claimed that 98% of children did not receive the dental care they deserved. The majority had decayed teeth, many of which were beyond saving, and very few had had any dental treatment. Thompson’s paper was well-received among NZDA members and came to the attention of Parliament, where it was printed and circulated as a parliamentary paper (3). However, awareness of the existence of an issue, such as children’s poor oral health, is no guarantee that an item will be placed on a Government’s agenda. The issue needs to be considered a legitimate one in which the Government feels it has a right to intervene, has the necessary technology, resources, money and personnel available, the infrastructure required, and the support of the public (4). Policy development usually occurs as the result of a multitude of factors that may include situational, structural, cultural, and environmental factors (5, 6). Furthermore, the CSDH framework notes that the socioeconomic and political context has a “powerful influence” on patterns of social stratification which, in turn, determine health status. Previous work on the determinants of health has paid little attention to the political context, however. The social determinants of health are shaped by Government policies; decisions made in the political context will impact on health and health inequalities but are themselves driven by a variety of political, economic, and social forces (2). In the case of the SDS, its establishment, and the form it took, was very much influenced by not only the political conditions of the era but also social and economic factors.

**Figure 2:**
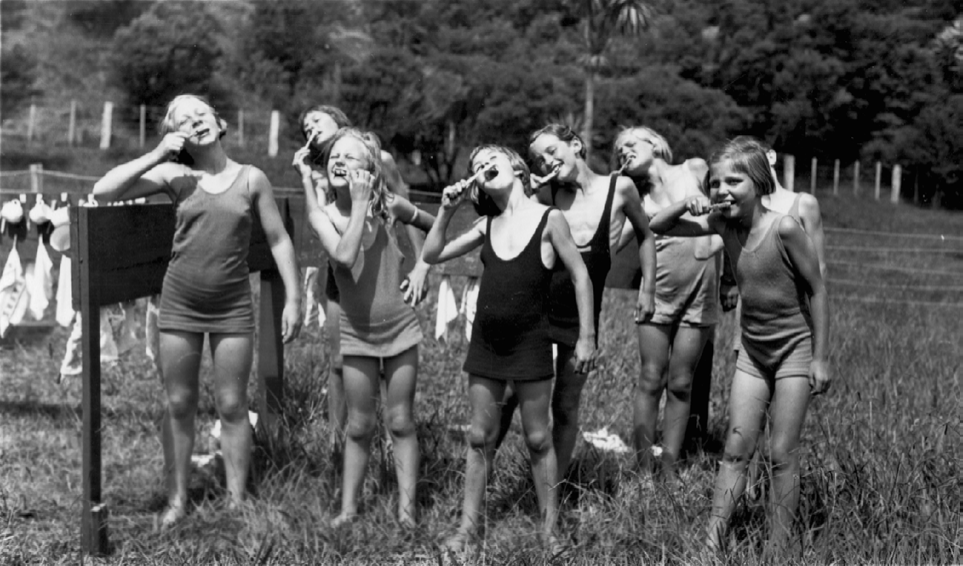
Toothbrush drill at a Health Camp [Hocken Collections, Uare Taoka o Ha¯kena, University of Otago Library (Archives Reference: AG-007-007-018/001)]. New Zealand’s Children’s Health Camps were founded in 1919 for primary-school-aged children who had health issues, such as malnutrition and tuberculosis (12). Nowadays, the Health Camps are more likely to cater for children needing help with social skills, “time out,” or respite care (13).

Lobbying by dentists and the NZDA for a state dental service for children came at a time when New Zealand’s Liberal Government (1890–1911) was already engaged in an extensive program of social reform. Its role in the economy and provision of public welfare were expanding rapidly (7), and New Zealand was developing a reputation as somewhat of a “social laboratory” for the world (8). In terms of health, a Department of Public Health was established in 1900, and once progress had been made addressing issues such as sanitation, clean drinking water, vaccination, and tuberculosis, attention turned to the issue of children’s health (9). Concerns about national efficiency and racial fitness compelled the Government to intervene where children’s health was concerned. Children were now regarded as “social capital;” investing in their health would ensure the race, nation, and Empire of its continued success (10, 11). As a result, social policy became centered on child health and welfare. Accordingly, several new health initiatives emerged, including St. Helen Maternity Hospitals (1904), the Society for the Protection of Mothers and Babies (or Plunket as it became known) (1907), the School Medical Service (1912), Physical Education in schools (1912), Children’s Health Camps (1919) (Figure 2), and eventually the SDS (1921).

Dentists also harbored concerns about national efficiency and the effect of poor oral health on general health; they and their medical counterparts attributed many childhood illnesses to poor oral health (14–16). However, while NZDA members had a true “crusading zeal” to improve children’s teeth, their campaign for a state-funded dental service for children was also political and tied up in their move toward professionalism (17). By advocating for a state-funded service for children, staffed by registered, preferably university-educated dentists, the NZDA hoped to close that corner of the market to unregistered, unqualified “mechanical dentists” whose patient group consisted mainly of the poor, Māori, and children. Lobbying for a service for children would also, hopefully, enhance their professional status in the eyes of both the Government and the public (17, 18).

While the NZDA continued to lobby and meet with Ministers of Parliament, there was little progress on implementing any type of service before the outbreak of the First World War in 1914. The war, however, drew more attention to the appalling state of the nation’s teeth (17). A high percentage of recruits were rejected for service due to their poor oral health and many others required extensive treatment to become “dentally fit.” Many attributed this poor state of affairs to lack of attention to oral health in childhood (19). As a result, the NZDA turned their focus to the formation of the highly successful Dental Corps (17). Priorities were different now, and there was no money for a school service. Although their attention was now focused elsewhere, the NZDA continued to discuss schemes for children’s dentistry for, as A.M. Carter rather melodramatically stated in his NZDA Presidential address of 1916 (20):

…the war of the nations will end, and in our hearts we know Victory will be ours, but in the dental disease so rampant in our schools we have a more insidious foe, and one that has been far too long underestimated, and that is steadily sapping the vitality and lowering the stamina of our national life.

In 1917, Richmond Dunn, a NZDA member, suggested that a new profession of “dental nurse” be created. Employing female dental nurses would go some way to solving the problem of the shortage of dentists at that time but would also relieve dentists of the “child-work” that many of them found so “trying to the nerves.” Dunn, however, believed that the dental nurse should have more of a preventive focus, and not merely be used to repair “the ravages of disease.” New Zealand’s Plunket nurses had been successful in offering advice and service to mothers and their babies, and a dental nurse might prove similarly effective in caring for children’s teeth (21). Dunn’s idea gained the support and a committee of NZDA members subsequently met with the Ministers of Public Health and Education to put forward its plan for a school-based dental service for children. However, the Ministers considered the cost prohibitive and that it was not possible to implement such a plan in wartime (17, 22, 23).

After the war, the NZDA tried again, this time supported by several powerful allies in putting its case to the acting Prime Minister, the acting Minister of Finance, and the Ministers for Public Health and Education. The deputation was well-received, particularly because the Ministers appreciated the NZDA’s “splendid work” during the war (24). After some initial delays, the Government appointed four school dentists in 1919 to form the basis of a school service, with control of the Service eventually passed to the newly established Dental Division of the Department of Health which was, in turn, responsible to a combined ministerial portfolio of Health and Education (17). Colonel (later Sir) Thomas Hunter was appointed Chief Dental Officer but resigned shortly after, when he learned that Government had failed to consult the NZDA over his appointment. This was a political move, reflecting the NZDA’s desire to play a major role in policy. When Hunter eventually took up the role in late 1920, it was as the Director of the Division of Dental Hygiene within the Department of Health (17).

## The New Zealand SDS Takes Shape

**Figure 3:**
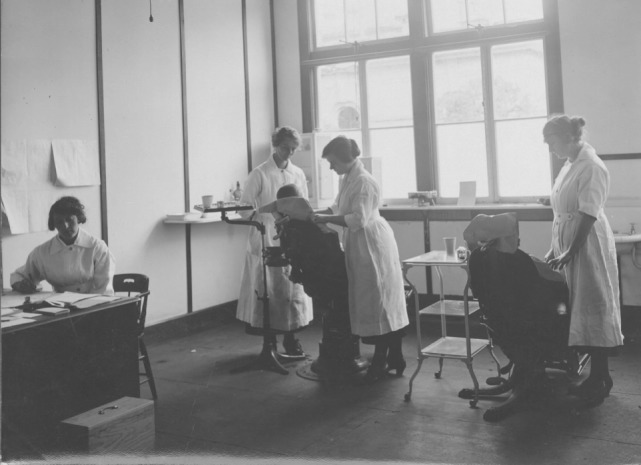
The extraction room, Training School, Wellington, 1922 [Archives New Zealand/Te Rua Mahara o te Kawanatanga, Wellington Office (Archives Reference: ABKI 667/1)]. Dental nurse trainees treated Wellington school children, with their oral health need being so great that an “extraction room” was set up. Two students each day would be assigned to the room and would spend the day extracting teeth.

In April 1921, the first “draft” of dental nurses commenced training in a Department of Health course, based in Wellington, the nation’s capital (Figure 3). Hunter had decided that 2 years’ training would be enough time to train the nurses to treat children’s teeth, mainly the “temporary” teeth. Dental nurses would be less expensive to employ and take less time and money to train than dentists. He also believed, no doubt influenced by stereotypical notions of women’s work and social norms of the time, that women were “temperamentally and psychologically more suited than men to deal with and treat the ailments of very young children.” Dental nurses were to be regarded as “auxiliaries,” especially trained for treating children, rather than “half-trained” dentists (25). Furthermore, while dentists may have felt threatened by this new role, marriage and children would prevent dental nurses from setting up their own practices because women in New Zealand’s Public Service had to stop working once they married (17, 25, 26).

Hunter was the driving force behind the SDS and was probably the most suitable person for the job at the time (17). He had served as President of the NZDA twice in its early years and had strongly supported the proposal for a dental service for children. Furthermore, during the war, he had been a very efficient Director of the Dental Corps (17). Hunter was passionate about his new role and committed to making a SDS, staffed by dental nurses, work. The model that Hunter developed, however, reflected the hierarchical power structures in health at the time. Doctors, usually white, middle-class, and male, ran the hospitals, and dentists from similar backgrounds would determine the direction the SDS took. The idea of a dental *nurse* would gain support from dentists, with the doctor/nurse relationship evoking ideas of a similar relationship between dentists and dental nurses. Nurses were expected to be obedient, disciplined and self-controlled, and had a place in paid employment but were no threat to men’s public roles (27). Very little consultation took place over the form the SDS would take, other than with members of the NZDA, who were determined to shape policy and the direction the SDS took, thus also protecting their own professional aspirations. However, although the role of the dental nurse had been defined as subordinate to that of dentists, the training program had a scientific basis, more so than nursing curriculums of the time, in which doctors dictated the level of knowledge required by nurses. The first Director of the School was Richmond Dunn, had been a science teacher, and he based the dental nursing course on the Dental School curriculum (28).

On graduation, the dental nurses were sent to work in school and community clinics. Schools were initially expected to establish a clinic and fund its ongoing maintenance, while the Department of Health supplied the dental equipment and the dental nurse (28). Clinics were many and varied; while some nurses went to purpose-built clinics, others worked in school classrooms, staff rooms, community halls, hospital buildings, shelter shed, and even school porches (29). The conditions were difficult, as was the treatment the dental nurses undertook. The majority of early dental nurses described the children’s teeth as “appalling” and “shocking” (30, 31). “They were incredible, those poor children with abscesses and the pain they must have endured… the extractions we had to make…” (30).

**Figure 4:**
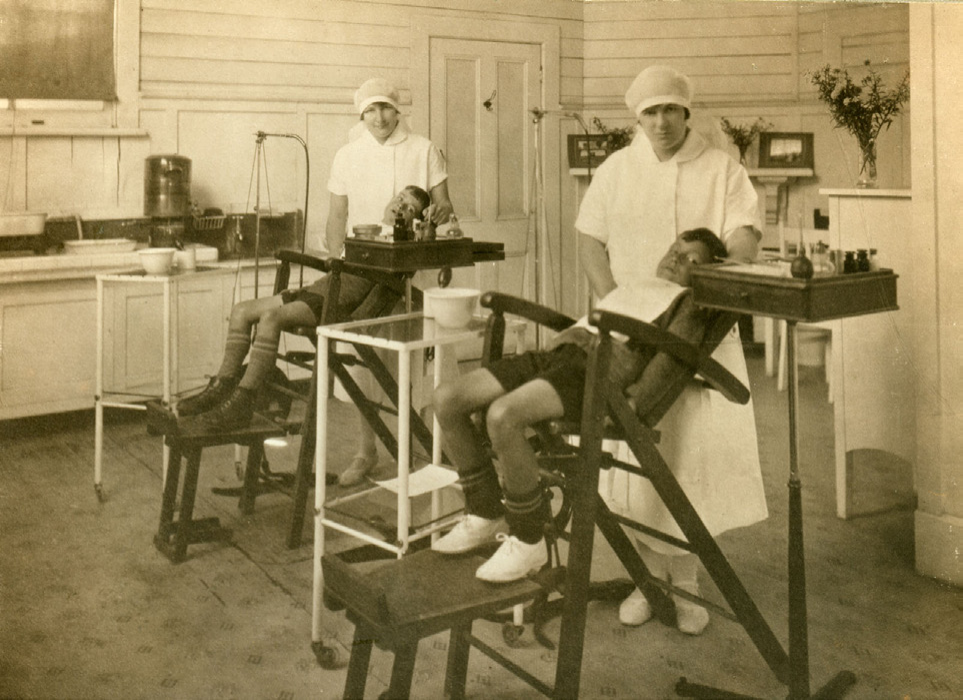
“Double clinic”—the interior of the Napier dental clinic before the earthquake [Archives New Zealand/Te Rua Mahara o te Kawanatanga, Wellington Office (Archives Reference 672/1)]. The dental clinic was destroyed during the 1931 Napier earthquake. Fortunately, the dental nurses and their patients escaped with very little injury (34).

By the end of the decade, more children were under the care of the Service, and working conditions for dental nurses were improving, with most clinics now situated in buildings jointly designed for the purpose by the Departments of Health and Education (Figure 4) (32, 33). In 1928, the Minister of Health announced that the Service would need 300 dental nurses in order to treat all school children. As the Service at this point only had 74 dental nurses and eight dentists, this was a significant expansion and demonstrated the support of the Government for the SDS (9). Hunter, however, noted that for those children who were already under care, there was still a lot of recurrent treatment. In his opinion, parents were not ensuring good oral health practices were being carried out at home and were placing “… the whole onus of caring for the teeth of their children on the State” (33).

## Depression, Welfare, and War

During the 1930s and 1940s, both social and economic conditions, and resulting policy decisions played a major part in the further development of the SDS. In particular, two major international events impacted on the SDS’s development, the first being the “Great Depression” and the second being World War II. While considered milder in New Zealand, the Depression, nonetheless, profoundly affected everyday life. The Government’s response to the country’s economic position was to appoint a “National Expenditure Commission” which recommended that the SDS not be allowed to expand further (28, 35). In the opinion of the Commission, there was an “…increasing tendency on the part of the community to look to the State for the provision of extra social services which had never in the past been regarded as the responsibility of the State.” Services that the State could afford in more prosperous times would have to be reduced or discontinued and the Commission recommended that the SDS not take on any new dental nurse students (35). However, rather than exclude students entirely, the Service reduced its intake between 1931 and 1935 (28).

Fewer dental nurses meant that arrears in patient treatment accumulated rapidly (28). To counteract the shortage of dental nurses, the Department of Health made the unusual move for the times of re-employing some married dental nurses (36). The decision was also made to maintain the 6-monthly recall for children already under care, and only extend treatment to new enrollments once this was under control (28). Parents were now charged a levy of up to five shillings for their child’s treatment and, once this was introduced, enrollments for care decreased to a certain extent. Parents could also apply for an exemption if they were unable to pay the fee and rising unemployment meant more exemptions were granted. For example, in 1934, of those enrolled for treatment at the training school clinic, approximately one-fifth did not have to pay (37, 38).

While the Commission made other recommendations, such as reducing the Government subsidy paid for schools to establish clinics, increasing the levy schools paid for their dental nurses, and even going as far as suggesting some dental nurses be dismissed, most of their recommendations were ignored (28, 35). This was most likely due to the fact that the Government was very aware that the SDS was valued by the public and calls for its expansion were increasing (28). This was somewhat of a political move on the part of the Government; however, although improving, children’s oral health was far from perfect and further downsizing the SDS would have been severely detrimental.

By 1935, the worst of the Depression was over and the SDS was able to once more increase its student intake. The SDS was to benefit further when the new Labour Government was elected at the end of 1935. By this stage, the service had approximately 50% of children aged up to 10 years under treatment but the Government wanted all children to be receiving dental care by 1940. Plans were put in place to double the number of dental nurses and to build a new training school (28). The intake of more students, however, brought no immediate relief to those working in the field. The shortage of staff placed limitations on how quickly the service could expand, and the closing of schools for several weeks due to a polio epidemic, placed further strain on the patient recall system (39). Despite the difficulties, figures documented toward the end of the 1930s show a steady increase in the numbers of dental nurses.^1^

In 1938, the Government introduced the Social Security Act, with hospital treatment, medicines, and general practitioner (GP) consultations all intended to be free of charge. The New Zealand Branch of the British Medical Association (BMA) successfully argued, however, against free GP visits, resulting in a part-subsidy/part-private funding arrangement (17). The Government had also consulted the NZDA on whether free dental care beyond primary school children should be included in the scheme. Discussion continued into the 1940s, with decisions being delayed somewhat due to the Minister of Health’s preoccupation with the BMA (17). John Llewellyn Saunders, by then Director of the Division of Dental Hygiene, further complicated matters, when he suggested that dental nurses could treat adolescents and adults (40). This did not go down well with the NZDA, who reminded Saunders that while the Government had previously approved extension of dental care, it was to adolescents only (28).

**Figure 5:**
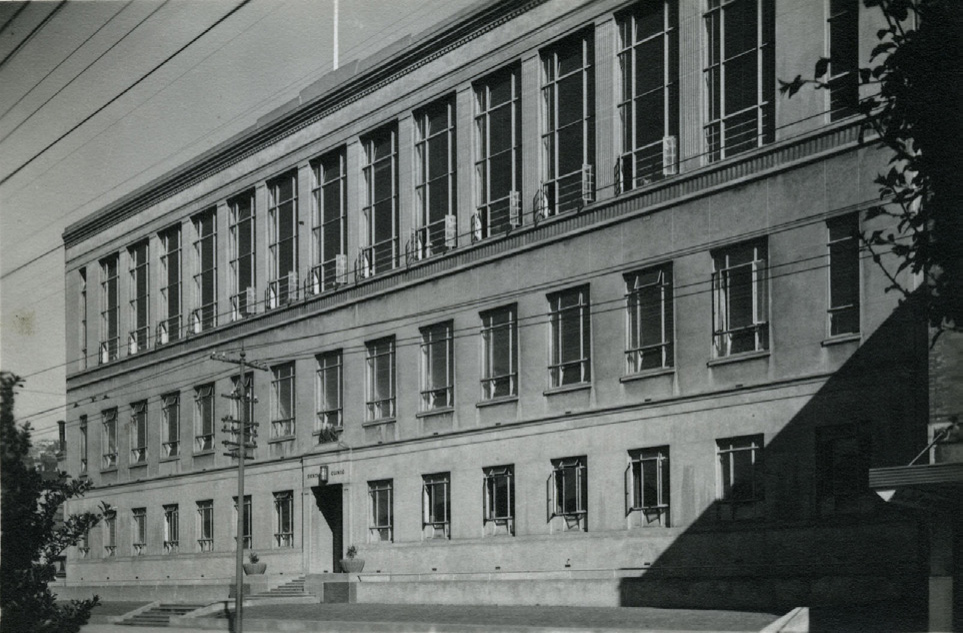
The Dominion School for Dental Nurses, Willis Street, Wellington, 1940 [Archives New Zealand/Te Rua Mahara o te Kawanatanga, Wellington Office (Archives Reference: ABKI 667/3)]. “In many ways its imposing structure made it appear like a temple of the welfare state. Solid, large and built to allow the most efficient use of space, it symbolised in architectural form the ideal of a benevolent, centralised State social service” (17).

The advent of war meant further delays to any decision about what form any possible state-funded dental care would take. For the SDS, war brought the slowing down of its clinic-building program due to the wartime control of labor and materials (41). Staff shortages were also an issue, with a higher loss of dental nurses through marriage during wartime. Although Saunders frequently lamented that war was slowing down plans for the SDS’s complete coverage of schools, good progress was made during this time. Despite the number of dental nurses only increasing slowly, the children brought under SDS care more than doubled (see text footnote 1). Dental nurses also started treating the older primary school classes, children aged 11–13 years, but could only accept these children after they had provided treatment for all preschoolers presenting for care. This was partly done to appease the NZDA, who were not only concerned about the amount of restorative work still being carried out and what they believed to be a lack of attention to dental health education and preschool oral health (42), but were also most likely also concerned about competition for patients, with an expanding SDS rapidly gaining favor with the public. The Government would also demonstrate its continuing commitment to the service and dental care for children by opening a new dental nurse training school in Wellington in 1940 (Figure 5).

## Oral Health Improvements and Inequalities

Policy decisions made by successive Governments determined the direction the SDS took and, in turn, impacted on children’s oral health. When the SDS was established, only children at state-funded schools were eligible to enroll in the SDS, and SDS policy dictated that dental nurses were to treat children in the junior classes first, then recall them regularly for care. There were issues with this policy; local communities were providing financial support for clinics but not all their children were being treated. Parents found it difficult to understand why their younger children could have free care but not their older ones (28). However, attempting to treat all the children would have meant the dental nurses would be restricted to “relief of pain” work only and, thus, be unable to bring children back for regular recalls. Furthermore, there would be no opportunities for preventive care or dental health education.

The progress of the SDS in treating dental caries was measured by the extraction-to-filling ratio. Initially, due to the very poor oral health of the children, dental nurses were extracting hundreds of teeth (43). A dental nurse from Dunedin, in later years, commented of her early experience in the SDS: “I did 1700 extractions in a year. Sometimes the pus would run down over your fingers. You’ve no idea what the mouths were like” (44). The extraction to filling ratio, however, improved over the SDS’s first decade, showing an almost fivefold decrease (Table 1).

**Table 1:** Number of extractions per 100 fillings (1921–1931) (37).

Year	Extractions per 100 fillings
1921–1922	114.5
1922–1923	103.3
1923–1924	79.7
1924–1925	72.6
1925–1926	67.2
1926–1927	62.8
1927–1928	56.3
1928–1929	52.3
1929–1930	37.2
1930–1931	25.5

Many Māori children attended “Native Schools” and could not initially enroll for care in the SDS but the SDS had selected four Māori students for training between 1925 and 1926 so that they could be “…trained for work amongst the Native children” (45). This indicated an awareness of a need for dental care for Māori children; Māori nurses were employed to care for Māori, particularly those in isolated areas and the intention may have been to do the same for oral health. By 1929, however, all schools (including denominational and private schools) were able to establish their own dental clinics (28).

At first, Māori children had better teeth than their European counterparts. The Department of Health reported in 1924 that European children had, on average, twice as many filled teeth as Māori children (46). The dental nurses also noticed that Māori children often had better teeth, with one commenting of the Māori children in Rotorua: “I have never seen such beautiful teeth… I can’t remember extracting a tooth from a Māori child” (47). In 1931, the Department of Health Annual Reports began to distinguish between the oral health of Māori and “White” children (48). These also confirmed that Māori children initially had better teeth (48–50). Their oral health, however, appeared to deteriorate rapidly when they adopted more “westernised” diets. By the mid-1930s, the Dental Officer for the Native Schools, Dr. Luke Rangi, observed that there was now very little difference between Māori and non-Māori teeth. “Both were equally bad.” He noted, however, that Māori children who lived further away from the “white centres of population” still had better teeth (38).

By the late 1930s, despite the “efforts” of dental nurses, school medical officers, district health nurses, and teachers, very few Native Schools had access to dental clinics. The “indigent Māori parent” and “apathy of the Māori people towards dental treatment” were considered to be the main obstacles to care (51). While Māori were stigmatized as not caring about their oral health by those in power in the Department of Health, in reality, Department decisions about where dental clinics were established meant that few Native Schools were located in areas near the growing network of dental clinics in the 1930s (52).

Access to health services for all New Zealanders in the early decades of the twentieth century was generally determined by their availability and affordability; however, services for Māori were further limited by cultural, bureaucratic, and geographical difficulties. In terms of oral health, Māori communities were less likely to have the financial means to establish clinics which also included paying for the dental nurse’s accommodation, non-technical equipment, cleaning, lighting, and a levy of £30 per dental nurse per year (52). Māori children who received no care at all quite possibly would have had worse oral health than those who were seen by the Dental Officer for Māori. The School Medical Service also did not have the resources or staffing to include all Native Schools in its service and District Health Nurses were in short supply. It often fell to the teachers at the Native Schools to offer health advice and care to Māori, including most likely dental advice. While differential access to healthcare could be considered a drawback of a developing public health service, nowadays this would be considered a form of institutionalized racism and a major factor behind health inequities. By the late 1930s, however, the passing of the Social Security Act (1938) and policies supporting universal access to health care improved Māori access to services. By 1941, the Government had abolished the levy school committees paid for their dental nurses, instead paying the committees an annual levy to cover running costs and encourage further development of dental clinics. In addition, parents no longer had to pay a fee for dental care as was the case during the Depression (53). The CSDH framework notes that population health is partly dependent on the type of welfare regime, with social democratic countries exhibiting significantly better population health status (2). In New Zealand, progress in Māori general health was facilitated by the policies and programs of the Welfare State (54). Although there are no specific statistics for oral health, it is likely that Māori oral health began to improve with better access to care. However, while the gap in health status between Māori and non-Māori narrowed during these years (as measured by mortality and morbidity); it was still very evident (54).

Overall, over this period, oral health continued to improve for children in the care of the SDS, with the extraction-to-filling ratio decreasing to 6.3 extractions per 100 fillings by the end of the war (55). Unfortunately, dental examinations of men entering the armed forces during World War II revealed that adult oral health was still very poor. Sixty percent of men had dentures and, of those with their own teeth, 80% required treatment (56). While free dental care was eventually extended up to the age of 16 years in 1947, by means of an adolescent dental service staffed by private practitioners contracted on a fee-for-service basis, free dental care was not extended to adults under the social security scheme (17). As a result, the establishment of the General Dental Benefit Scheme (as the adolescent service became known) appeared to merely shift the age at which New Zealanders developed oral health problems. Previously, on leaving the SDS at aged 12 or 13 years, and no longer having free dental care, children developed oral health problems during adolescence. Now that free care was available up until the age of 16 years, problems developed between 17 and 20 years of age. Affordability of care was neither mentioned by Saunders nor the NZDA, however, and neither were the other social determinants of health considered, such as employment or having money for housing and food, which were priorities during the Depression and War. Most of the blame for poor oral health was laid on young people or their parent’s “don’t care” attitude to oral health and to the prevailing New Zealand belief that problem teeth should be extracted and dentures were inevitable (57).

## The SDS and the “Baby Boom”

From the end of World War II up until the 1970s, the SDS struggled to meet its goal of providing care for all school children. In this era, social conditions played a major role in the further development of the SDS. Postwar labor shortages and a “baby boom” put pressure on many health services, as well as preschool, primary, and tertiary education. Rather than achieving full coverage of all primary schools as previously predicted, the SDS found it difficult to keep up with the number of children being born, with staff shortages a major factor.

During the war, women had been encouraged to work; all women between 18 and 40 were required to register for “man-powering” but when the men returned from war, the Government’s rehabilitation program promised them a return to full employment (26). This created an exodus of married dental nurses from the SDS who resigned when their husbands came home. Although the numbers of dental nurse students in training had increased, the overall numbers in the field decreased (55), and other professions, such as the teaching and nursing, were also facing shortages (26). As a result, the Government promoted recruitment of married women and allowed them to be permanently employed. While postwar policies encouraged women to return to their homes, the State undermined domesticity by encouraging women to re-enter the workforce (26).

The labor shortages were exacerbated by the “baby boom.” The baby boom in New Zealand has been described as having two distinct phases; the first phase in 1945–1946 being a “family size catch-up,” following low fertility rates during the Depression and the war, while the second phase, in which large family sizes became the norm, lasted until the early 1970s (58). The SDS developed innovative recruitment campaigns, as did teaching and nursing, to recruit young women to their professions and encourage married women back to work. This was further hampered, however, by the fact that the birth rate during the Depression had been low; therefore, the school-leaving cohort was small (26). To further deal with staff shortages, Saunders had negotiated an “emergency plan” with the NZDA. Upper primary school classes could be transferred to “general dental benefits” and be treated by dentists, thus enabling the dental nurses to concentrate on the younger patients, including the ever-increasing preschool roll (28).

**Figure 6:**
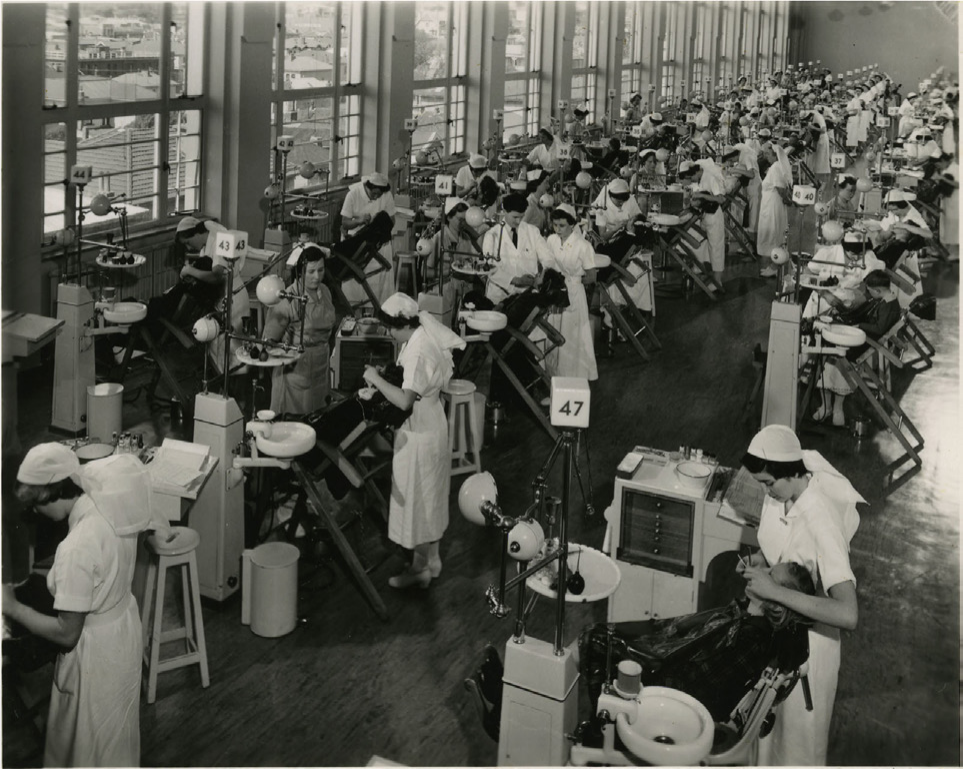
The Dominion School for Dental Nurses, Wellington—showing many students and staff, early 1950s [Archives New Zealand/Te Rua Mahara o te Kawanatanga, Wellington Office (Archives Reference: ABKI 667/3)].

The SDS continued to receive support from successive Governments in the face of its staff shortages. Two new training schools were built in the 1950s, one in Auckland and one in Christchurch (59, 60), and further expansion to the training occurred in the 1960s when the service established a number of “section clinics,” built on primary school grounds (61). The student intake reached a peak in 1964 and 1965 with over 270 students being enrolled each year into the program (Figure 6). By the end of the decade, there were 1,334 dental nurses in the field and by March 1970 the number of children under the care of dentists had been reduced from 16,949 to 9,159 (62). It was anticipated that the patient group would soon include, for the first time, every primary school child in the country, and many of the preschoolers (63).

## Efforts to Improve Oral Health

While the SDS’s main focus seems to have been “full coverage,” efforts were made to further improve its effectiveness, particularly in preventing dental caries. From the early days, as well as providing oral health instruction at the chairside and dental health education in the classroom, dental nurses carried out various forms of preventive treatment, including cleaning teeth, applying silver nitrate to arrest early carious lesions and prophylactic odontotomy, which involved filling the deep fissures of permanent molars in order to prevent decay of those fissures (64). The introduction of fluoride to the SDS, however, was perhaps of most benefit in preventing caries. By 1950, dental nurses were applying fluoride to children’s teeth (65) and in 1966, evaluations found that this method of fluoride application significantly reduced caries in children (66).

New Zealand was one of the first countries to instigate water fluoridation, supported by a parliamentary system that held political and fiscal responsibility for decisions on the health and welfare of its people. Fluoride was first introduced, initially on a trial basis, into the water supply in the town of Hastings in 1953. Results of the trial showed that after 16 years’ continuous fluoridation, for children aged 13–15 years caries prevalence was reduced by 50%, and for 16-year-olds, by 40% (67). By this time, 60% of New Zealand’s population was on a reticulated water supply using fluoridated water and continued water fluoridation meant that dental nurses were able to handle higher roll numbers (68, 69). Where fluoridation had been in operation for some time, the amount of treatment required was reduced. For example, in March 1970, 14,845 more children were under treatment than the previous year but the total fillings required fell by 66,481 (62, 70).

There were, however, few surveys done on the general oral health status of New Zealanders in this era. Oral health surveys of army recruits in the 1950s revealed that there had been a reduction in their loss of teeth (71), while a survey of young adults carried out in 1962–1964 found that the DMFT for 15- to 19-year-olds was 16.73 with 3.2 decayed and 0.88 missing teeth, suggesting a level of unmet need and quite a high level of tooth loss for this age group. Although “race” was collected from those surveyed in 1962–1964, the findings do not differentiate by ethnicity or socioeconomic status so it is not clear whether there were inequalities in oral health during this period (72). When looking at general health, however, there was an awareness at the time that there were inequalities in health between Māori and European but little attempt was made to quantify differences until the late 1950s, most likely due to the fact that Māori health policy before that promoted assimilation (9). In April 1960, the Department of Health published “Māori-European Standards of Health” which “… [indicated] very clearly that the health standards of the Māori [were] very low in comparison with the European” (73). Given that there were inequalities in other health areas, it is likely that this was also the case for oral health.

Later in 1960, J.K. Hunn’s “Report on the Department of Māori Affairs 24 August 1960” examined issues such as land, housing, and education, the outcome of which led to a commitment to eliminating differences based on inequality or discrimination. This was perhaps the first occasion where there would be official acknowledgment that social or structural factors (such as education, occupation, and income) play a part in determining health. As a result of this report, the Department of Health acknowledged that “…adverse environmental conditions give rise to consequential disadvantages, in health and otherwise, for many Māoris (*sic*) and there is little that the [Māori Health] committee can do to alleviate these circumstances” (74). The 1950s were, in fact, a period of great change for Māori, as this was a period of Māori migration into the cities to take advantage of the new employment opportunities that became available after World War II. While for some, this led to wider educational and employment opportunities; for others, the cultural and social dislocation led to issues, such as alcohol and drug abuse, violence and crime, and physical and mental health issues. With the Māori workforce being mostly unskilled and in lower-paid employment, they were more vulnerable in times of economic downtown, which in turn had an effect on health. Fewer educational qualifications led to lower-income jobs or unemployment, resulting in lower standards of housing and health, including most likely oral health, given that dental treatment over the age of 16 years had to be paid for by the individual (75).

Efforts made to improve inequalities in health in this era (1950s, 1960s) focused on issues, such as infant mortality, health of Māori mothers and infants, and tuberculosis, with Māori health policy being incorporated into public health and hospital policy. By the end of the 1960s, the official Department assessment of “Māori health trends” was optimistic; however, other commentators had different views. The Editor of the *New Zealand Medical Journal* described the emphasis on Māori health as “…our particular problem with underprivilege in the midst of plenty” (9).

## The SDS: From Celebration to Critique

**Figure 7:**
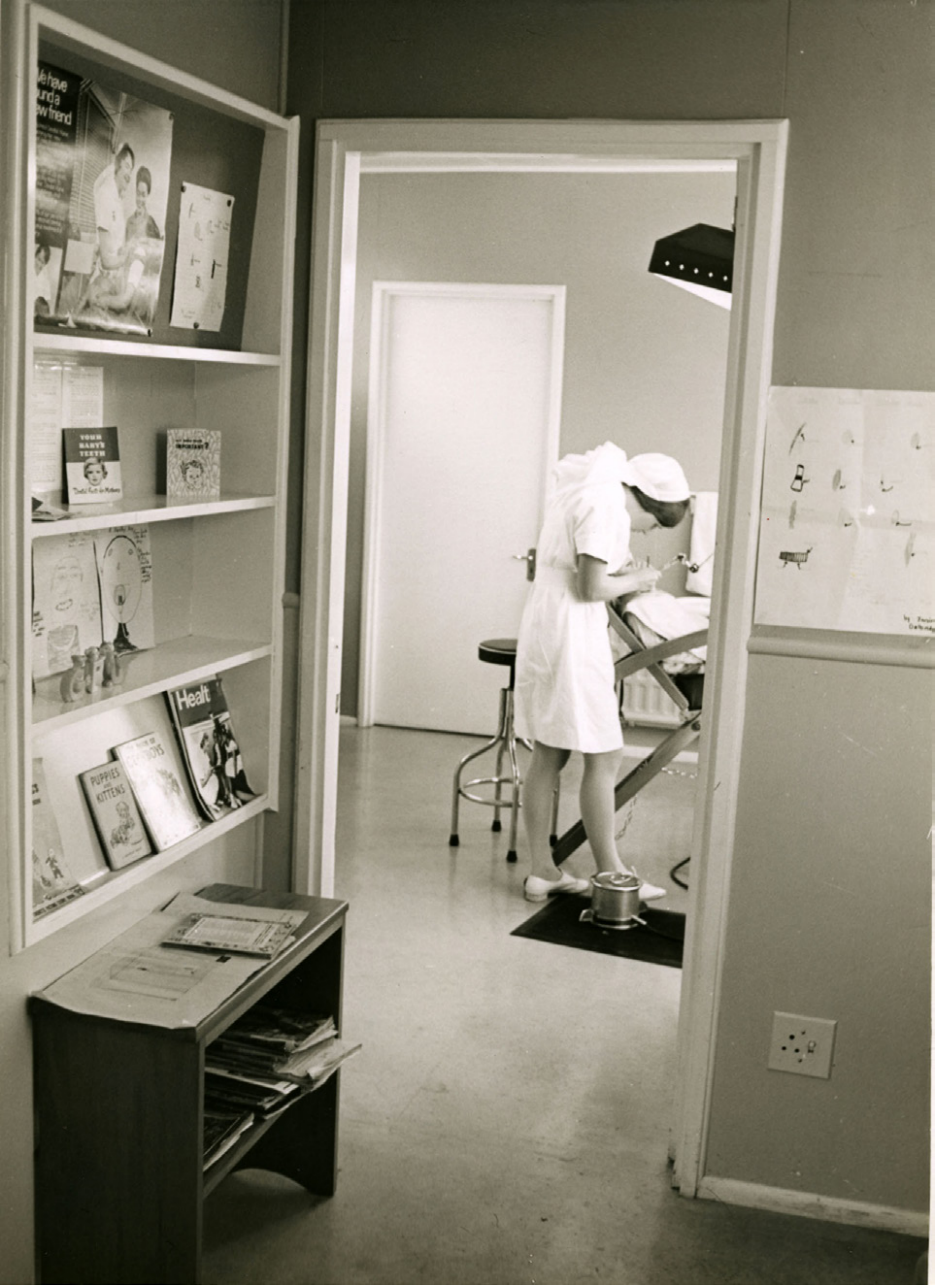
Dental clinic and dental nurse, 1970s [Archives New Zealand/Te Rua Mahara o te Kawanatanga, Wellington Office (Archives Reference: ABKI W4078 667/3)]. Cuts to health funding from the late 1970s meant that the School Dental Service had difficulties in keeping up-to-date with modern procedures, e.g., the introduction of block anesthesia and radiography, and less money was available for new dental materials and modern equipment. Poor working conditions and a lack of career progression would lead to dissatisfaction within the workforce. What had once been an innovative service was rapidly becoming outdated (81).

The 1970s started on a high note, with the SDS celebrating its Golden Jubilee in 1971. Messages of congratulation were received from all quarters celebrating the progress of the Service (76–79). By this stage, New Zealand was considered by many countries to be a world-leader in providing dental services for children. Progress continued into the mid-1970s, with the SDS finally achieving its goal of “full coverage” for all primary school children, as well as approximately 65% of preschoolers, by 1976 (80). Some 1,341 school dental nurses were working in 1,297 clinics, taking care of 582,964 preschool and school-age children (Figure 7) (76). The extraction-to-filling ratio had decreased further and was now only 2.8 extractions per 100 fillings (78). However, as the decade progressed, the dental profession would become aware that there were still challenges ahead in regard to improving the oral health of New Zealanders.

In 1973, the World Health Organization (WHO) conducted the International Study of Dental Manpower Systems (ICS I) in several countries, including New Zealand. This study found that 8- and 9-year-olds and 13- to 14-year-olds in the Canterbury region had a low unmet need for restorative treatment, which indicated that the SDS was successful in meeting their treatment needs, but that they had heavily filled teeth (82, 83). The 13- to 14-year-olds who had enrolled with the SDS at the age of 5 years in 1965 would receive, on average, a total of 37 restorations (in both deciduous and permanent teeth, and including filling replacements) (71). There clearly was a need to concentrate more on the prevention of caries rather than control of caries through fillings.

Some 36% of the 35- to 44-year-old New Zealanders in the WHO study were fully edentulous. New Zealand had the highest percentage of adults from this age group with no natural teeth when compared with the other countries surveyed (82). The Editor of the *New Zealand Dental Journal* claimed that before the survey, “New Zealand dentistry tended to be rather smug” and had boasted to the rest of the world that they had “…the greatest SDS the world had ever seen.” “The alarmingly high level of edentulousness in New Zealand shook [them] all out of [their] smugness” (84).

The findings of the WHO survey prompted the New Zealand Dental Research Foundation to carry out its own Survey of Adult Oral Health and Attitudes to Dentistry (SAOH) in 1976. This study had similar findings to the WHO survey. By the age of 20 years, New Zealanders had approximately half of their teeth decayed, missing, or filled. By 40 years, this figure had risen to 75% and by 65 years, to 96%. For those under 20 years of age, there had been a reduction in dental decay in more recent years but periodontal disease was an issue, and oral hygiene was inadequate at all ages. One-third of those over 20 did not have their own natural teeth (85, 86).

Further analysis of the WHO survey revealed that the state of adult oral health was also dependent on socioeconomic status and whether New Zealanders lived in rural or urban areas. Those in lower socioeconomic groups were less likely to visit dentists and more likely to have dentures due mainly to the cost of dental care (82). Peter Davis, a sociologist involved with the SAOH, observed that the SDS did not “…eradicate the ‘social class gradient,’ nor did it reduce the rural-urban difference” (17). The SAOH had similar findings in regard to socioeconomic status and this survey also differentiated between ethnic groups, finding that Māori were more likely to have poor oral health than their European counterparts. This survey was perhaps the first to note that “dentally advantaged and disadvantaged” groups existed in New Zealand society (86).

## Child Oral Health Inequalities Revealed

The findings of the previously mentioned surveys, initially presented in a Symposium at the Dental School, led to a workshop of key stakeholders being held in Rotorua in 1978, from which several recommendations were made (17). Of relevance to the SDS were targets put in place to reduce dental caries within the next 10 years, which included reducing the dmf for 5-year-olds to 3 and the DMF for 12- to 13-year-olds to 5. The goal for the percentage of caries-free 5-year-olds was set at 50% (then 34%), while the goal for 12- to 13-year-olds was 20% (then 2.4%) (87).

The SDS acknowledged that their diagnosis of caries required reassessment and dental nurses were now actively discouraged from restoring early carious lesions, with direct fluoride treatment of early carious lesions being advocated (88–91). A 30-min “preventive appointment” was introduced to the SDS involving early detection of disease, clinical preventive care, and chairside counseling (92). Targets set in place for reducing the numbers of fillings resulted in a 55% reduction between 1977 and 1981 (91). In 1980, a new ratio was introduced to evaluate the increased emphasis on prevention, that of fillings in *permanent* teeth per child. Using this ratio, a retrospective examination of record revealed that in the years 1976–1981, a 64% reduction in fillings had occurred (91).

Surveys carried out by the Health Department on 12- to 13-year-olds in 1977 and 1982 showed that while DMFT had decreased between the years surveyed, non-European children and children from non-fluoridated areas were likely to have poorer oral health, with their DMFT scores, on average, 15% lower than their European and fluoridated area counterparts (93) (Table 2). Surveys carried out on 5-year-olds also demonstrated a decrease in dmft over the period; however, dmft was “substantially higher” for non-European children (94) (Table 2). These surveys showed that 47% of preschoolers were enrolled with the SDS by the age of 3 years and 87% by the age of 5 years but also revealed that non-European children were less likely to be enrolled in the SDS (94). Oral health data from the Dunedin Multidisciplinary Health and Development (DMHD) study revealed similar patterns. The data for children at age 5 suggested that there was a socioeconomic gradient in dental caries between children living in fluoridated and non-fluoridated areas, with the gradient being more obvious in children in non-fluoridated areas (95).

**Table 2:** The dental caries experience of New Zealand children in 1977 and 1982 (93, 94).

	European	Non-European	Fluoridated^a^	Non-fluoridated	All children
**DMFT of 12- and 13-year-old children**					
1977	6.7	8.6	6.3	7.8	7.0
1982	3.6	4.3	3.6	4.2	3.7
**dmft of 5-year-old children**					
1977	3.3	6.1	3.4	4.2	3.7
1982	2.2	3.9	2.3	3.0	2.6

*^a^Lifetime fluoridation*.

The likely reason that oral health inequalities had gone unnoticed prior to the 1980s was partly due to the Division of Dental Hygiene’s method of monitoring oral health, which did not distinguish between ethnicities. The Department of Health was probably not fully aware of this developing child oral health issue. Furthermore, although some attempt had been made to quantify differences between Māori and European general health since the late 1950s (9), oral health does not appear to have been considered. As a result of concerns about “worrying statistics,” the Medical Research Council of New Zealand had commissioned Dr. Eru Pomare to undertake a study of Māori Health Standards covering the years 1955–1975. Published in 1980, Pomare’s research confirmed that Māori health was worse than that of Pākehā^2^ (European) for many conditions, such as coronary heart disease, cancer, diabetes, asthma, rheumatic fever, and mental health issues (96). That this report (or Pomare’s second report reviewing the years 1970–1984) does not mention oral health indicates that oral health was not always considered to be a part of general health (96, 97). While there may not have been much information available on the differences between Māori and Pākehā oral health at the time of publication of the first report, results of several surveys would have been accessible by the time the second report was published.

It is also possible that the SDS’s prior emphasis on providing “full coverage” of dental care, and the focus on reducing dmft/DMFT from the late 1970s, came at the expense of evaluating thoroughly the care already being provided. This was evident in a paper written in 1984 to explain evaluation in dental public health in New Zealand; while it noted improvements in oral health as a result of the efforts of the SDS in response to the oral health surveys of the 1970s (ICS I and SAOH), it failed to acknowledge that these surveys, and the more recent Health Department surveys for 5-year-olds and 12- to 13-year-olds, had identified inequalities in oral health between groups of New Zealand children (91). By 1986, the SDS had achieved the goals set at the Rotorua workshop and also those of the WHO set in 1981^3^ (91, 98, 99). When the WHO carried out the second part of its International Collaborative Study (ICS II) in 1988, New Zealand showed a dramatic improvement in oral health for all age groups but ICS II also revealed socioeconomic and ethnic differences in oral health status, with Māori and those in lower socioeconomic status groups having poorer oral health (100).

It is very likely, however, that socioeconomic and ethnic inequalities in oral health became more apparent in the late 1970s and 1980s because this was a time of great change for New Zealand. Economic conditions and resulting policy decisions would have a major effect on health during this period. The previous two decades had been a period of record economic growth which both supported New Zealand’s Welfare State and enabled the Government to further extend the SDS. During the 1970s, however, economic growth slowed dramatically and, by the end of the decade, restraints on Government expenditure, including health, had been imposed. New Zealand was no longer able to afford its Welfare State. Under a National Government (1975–1984), led by Prime Minister Robert Muldoon, New Zealand experienced an economic setback described by one commentator as the “…most prolonged postwar recession amongst the industrial capitalist countries” (7). Inflation was high, unemployment rose, and inequalities in income increased. All of these impacted negatively on health, particularly among the Māori and Pacific communities, who were more likely to be unemployed and earning less.

Pomare’s second report (1970–1984) showed that while economic conditions affected health status for Māori, cultural and social conditions also had an impact. Pomare observed that: “Māori people [were] grossly disadvantaged socially, economically and culturally” (97). They were more likely to have fewer educational qualifications, be over-represented in prison, living in poor housing, all of which impacted on both physical and mental health. The majority of Māori occupied the lower socioeconomic bracket and this, combined with cultural factors, was considered among the most important reasons that Māori experienced more ill health. Māori were less likely to have money available for medical care, nutritious food, and adequate housing. Where oral health is concerned, the SDS may have been free, but Māori may have been less able to access it or pay for transport to clinics. Culture and self-worth was also considered an essential component of health for Māori and Pomare noted that many Māori might not access available services due to cultural barriers (97). This could also explain why Māori would not necessarily attend a free SDS, staffed by predominantly Pākehā female dental nurses, and run within an essentially mono-cultural health system.

Despite the increase in inequalities in health during the 1980s, this period would mark a turning point in terms of health policy for Māori. In 1984, Māori health advancement was identified as a priority at two Māori Health Hui (meetings), with Māori also expressing a desire to provide health services to their own people.^4^ While Hunn’s 1960 report had been credited with moving Government policy from assimilation to integration for Māori, the 1980s would see a commitment to biculturalism in policy (101). For the Department of Health, this meant taking steps to include Māori perspectives in its policies and practices, as well as formally acknowledging the relevance of the Treaty of Waitangi to health provision (9). Furthermore, legislation would ensure that the Crown, the Government, and the health sector could easily consult with Māori (101).

## The End of an Era

The early 1980s had seen a downsizing of the SDS; as well as requiring the SDS to reduce salary costs (102), the Minister of Health instigated a review of dental nurse training that resulted in the closure of the Auckland and Christchurch training schools. While the review could be partly attributed to the need to save money, there were other factors to consider as well. The birth rate was now in decline and the average number of fillings per child had fallen from 5.0 in 1965 to 1.8 in 1979. In addition to this, there were 400+ dental nurses on either special leave or former dental nurses wanting to return to work (103). However, more significant change was ahead for the SDS.

While in power, the National Government (1975–1984) had proposed that 14 regionally-located, locally-elected Area Health Boards (AHBs) be established with the intention being to amalgamate the existing Hospital Health Boards and “…integrate their (curative) functions with the Department of Health’s (preventive) district health offices” (104). The subsequent Labour Government (1984) chose to continue with this concept and introduced a population-based funding formula to make the most efficient use of an already-reduced health budget, and to shift the previous focus from hospitals to include epidemiological factors and public health programs (104). The Department of Health was subsequently restructured; with most administrative and service delivery functions transferred to the AHBs, it now had a policy development and implementation role (105).

The move to AHBs has been described as the “end of an era” for the nationally directed SDS (105). Responsibility for the management of the Service was now delegated to the 14 Boards, in effect replacing a centralized Service with 14 independent regional SDSs (105). In a time of financial restraint, it may have been tempting for AHBs to divert money away from the SDS to other Board services; however, on being appointed Minister of Health in 1989, Helen Clark opted to retain responsibility for primary care and general practice services within the Department of Health (104). The Department would remain accountable for SDS policy and funding. Furthermore, the Minister viewed the SDS as having a “…central role in oral health promotion and disease-prevention methods,” and dental nurses able to extend the areas within which they could work, based on population needs (106). Moreover, acceptance as the SDS as a public institution was embodied in the widespread (and somewhat pejorative) use of the term “murder house” (107).^5^

## The Early 1990s Attack on the Welfare State

**Figure 8:**
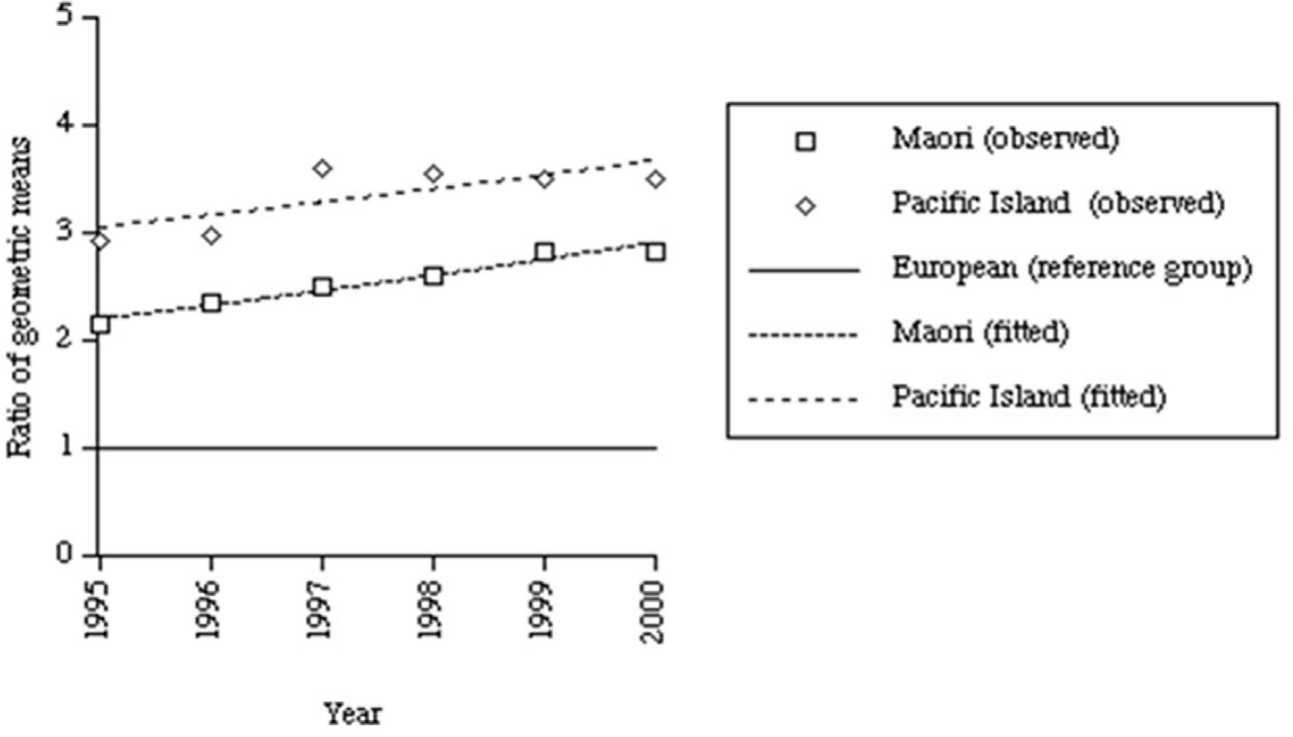
Evidence of widening in ethnic inequalities in deciduous dentition caries experience among Wellington 5-year-olds after the early-1990s social and economic policy “reforms” [from Thomson et al., (110); reproduced with the kind permission of the *Australian and New Zealand Journal of Public Health*].

With the passing of time, it became apparent that the capacity of the SDS and other dental clinical services to prevent oral diseases was rather limited and that the major determinants of poor oral health lay beyond the reach of those services. This was most notable in a steadily worsening lack of control over the cariogenic environment, together with deliberate, neoliberal-inspired social and economic policy decisions that were taken in New Zealand in the early 1990s. These included cuts to welfare benefits in 1990, the introduction of “market rents” for State housing in 1991, and the introduction of the Employment Contracts Act, also in 1991, which, in favoring individual negotiation over collective bargaining, essentially depowered the trade union movement. Neoliberal policies such as these lead to social inequality, which in turn leads to inequalities in health (108). Higher income inequality is also likely to affect social cohesion, another important determinant of health (109). In New Zealand, the Gini (income inequality) coefficient increased from one of the lowest in the OECD to one of the highest by the mid-1990s. Māori were much more affected by these structural reforms, being more likely to work in the laboring, manufacturing, and less-skilled service industries, sectors that bore the brunt of the reforms. Unemployment rose from 11% in 1986 to 25% in 1992 for Māori, while European unemployment peaked at 8%. Poverty among Māori households rose from 14 to 41% compared to an increase of 8 to 17% for Europeans (108). Overall, these ill-advised social policy initiatives resulted in many more New Zealanders living in poverty and caused a rapid widening of ethnic inequalities in child oral health over the subsequent 5 years (Figure 8) (110).

**Figure 9:**
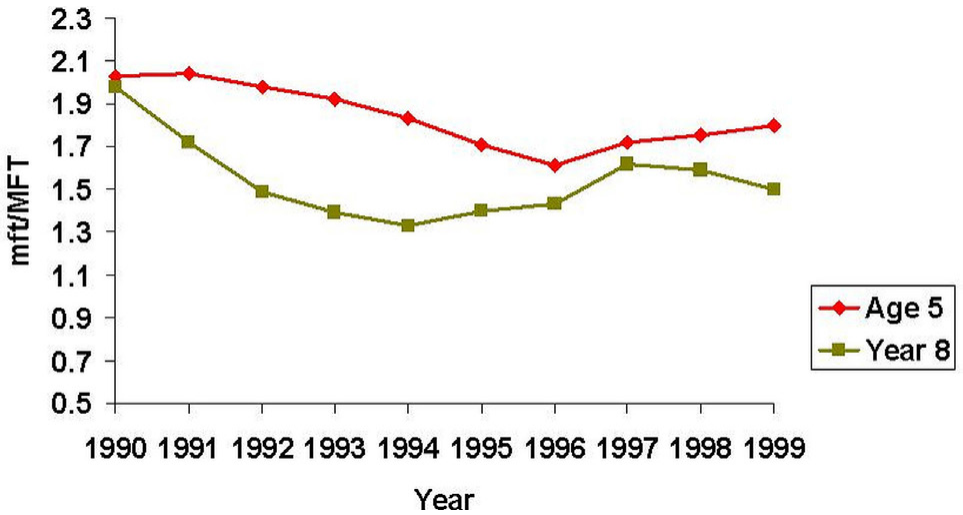
Trends in dental caries severity among 5-year-old and year 8 (12- to 13-year-old) children during the 1990s (111).

During this time, New Zealand also underwent a series of health “reforms,” aimed to reduce the role of the State in health care, and increase efficiency, choice, and responsiveness for consumers. These changes had a negative impact on school dental services, however, including redundancies for dental nurses (now called dental therapists). Increased workloads for dental therapists, along with mobility between clinics, led to a focus on treating caries with little time for preventive care and health promotion. SDS data for the 1990s show that while the mean mft scores for 5-year-olds fell between 1990 and 1996, they began to rise after that. Similarly, the mean MFT scores for year 8 (12- to 13-year-old) children declined from 1990 to 1994, then increased until 1997 before leveling off over the next 2 years (Figure 9) (111). One positive outcome of the 1990s health reforms, however, was an increase in Māori Health Providers who were able to secure contracts to provide services for Māori, and those included oral health services. These providers are owned by Māori, are operated under kaupapa Māori (Māori ideology and practice), and offer a whānau ora (family health) approach to care (101, 104, 112).

## Good Oral Health for all, for Life

Having suffered through a series of market-oriented “reforms” that emphasized efficiency over equity as a system goal (113), New Zealand had a change of Government in 1999. The new Labour Government introduced further changes, this time to improve disadvantaged social groups’ access to health care services. However, the neoliberal economic models that had gained global ascendancy during the 1980s and 1990s created obstacles to many of these policy actions. The New Zealand Government, like others of the time, embraced the principle of intersectoral action to address the broader social determinants of health. Under the banner of “Health for All,” the overarching goal was to improve the health of disadvantaged groups and reduce inequalities in health, and the Government would make this a priority within the health sector and wider policy arena over the next decade (114). However, some policies were controversial, for example, the “Closing the Gaps” affirmative action strategy pitched at Māori was widely criticized as showing favoritism to Māori at the expense of other equally disadvantaged groups.

The New Zealand Health Strategy (2000) specified “improving oral health” as one of 13 health priorities and one of 12 priorities for Māori health, thus demonstrating the Government’s acknowledgment that poor oral health was still a major issue for New Zealanders (114). A report to the Minister of Health in 2002 further confirmed that although child oral health had improved over the years, oral health inequalities were significant for Māori and Pacific children and adolescents, and children and adolescents from low socioeconomic backgrounds (111). Moreover, research on the oral health of participants in the DMHD Study found that although free dental care during school years reduced the effect of SES inequity, “profound” socioeconomic differences re-emerged by the age of 26 years, and high disease experience early in life led to greater disease experience in adulthood. Recommendations for tackling these oral health inequalities included targeting the social determinants of health and developing more suitable oral health services (115). Unfortunately, the previous years of reduced funding had left a SDS with poor working conditions and a dissatisfied staff with poor morale, this being further confirmed by national SDS facilities and workforce reviews (116–118). These reviews, along with the “Improving Child Oral Health and Reducing Child Oral Health Inequalities” strategy (111), would become the basis for a new strategic vision for oral health, “Good Oral Health for All for Life” (GOHFAFL). This was an opportunity for the Government to re-orientate the delivery of publicly funded oral health care in New Zealand (119). It represented a change in the assumptions that underlie the delivery of oral health care and required oral health to be placed in the context of other health strategies.

Good Oral Health for All for Life comprised seven “action areas,” these being the reorientation of the child and adolescent oral health service, reduction in inequalities in oral health and access to oral health services, promotion of oral health, building links with primary health care, building an appropriate oral health care workforce, development of oral health policy, and ongoing research, monitoring, and evaluation. Each District Health Board (DHB) was to develop a plan (business case) to suit the needs of its community, with a focus on prevention, early access for care, and a seamless service that provided care for children from birth to age 18 (Table 3). Significant Government investment funded new community-based clinics and mobile dental vans, complete with new equipment and modern technology. Dental therapists no longer worked in isolation; they now worked as part of a team, aided by dental assistants and administrative staff (119).

**Table 3:** A comparison of oral health approaches [adapted from Ref. (119)].

School Dental Service	Community Oral Health Service
An emphasis on treatment	An emphasis on prevention and early intervention
A division between oral health and general health	Oral health is integrated into general health frameworks
District health boards (DHBs) provide services	There is a mix of service providers, including DHBs, primary health organizations, Ma¯ori and Pacific providers, and non-governmental organizations
School-based dental services for children	Community-based dental services for children, with the potential to expand to adolescents and low-income adults
Separate funding for child and adolescent oral health services	Funding that allows flexibility of service program design
An emphasis on primary school years	An emphasis on preschool and early primary school years
Clinicians work in isolation	A team-based approach to oral health—dentists, dental therapists, and dental assistants work together
A small Ma¯ori and Pacific oral health workforce	A workforce more representative of the ethnic diversity of New Zealand
Pressure on secondary services	Greater capability at the primary care level, with secondary services focused on patients who cannot be managed by primary care

**Figure 10:**
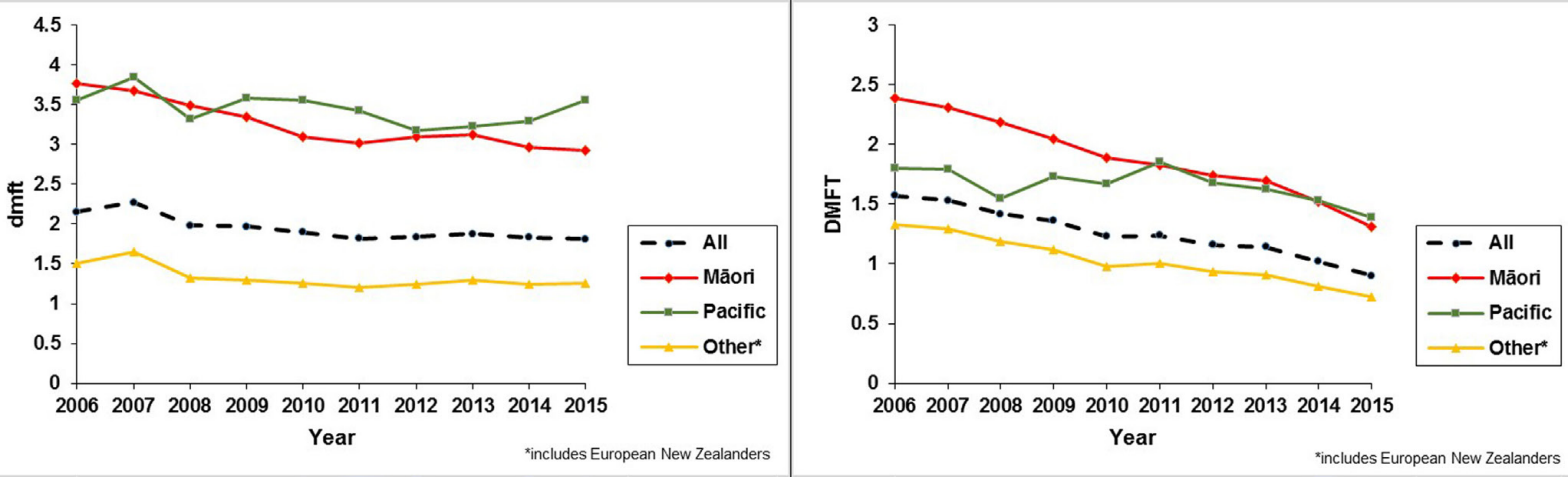
Dental caries experience of 5-year-old and year 8 (12- to 13-year-old) New Zealand children from 2006 to 2015. The data in these line graphs represent cumulative dental caries experience among 5-year-olds (dmft) and 12- to 13-year-olds (DMFT) for all of New Zealand over a 10-year period (120).

New Zealand’s 2009 Oral Health Survey showed that large improvements in oral health have occurred for children since the 1980s; the proportion of 12- to 13-year-olds who were caries-free almost doubled between the time of the last oral health survey in 2008 (28.5%) and 2009 (51.6%). DMFT has also significantly decreased for this age group (from 2.4 to 1.3). However, the 2009 survey also found that significant disparities in oral health status and access to care still existed for Māori and Pacific children and adolescents (1). In recent years, COHS 5-year-old and year 8 average dmft/DMFT and caries-free data also suggest that, overall, oral health for New Zealand children is improving but that inequalities in oral health persist (Figure 10) (120, 121). For Pacific Island 5-year-olds, there is an apparent worsening of oral health but it is not yet known whether this is a longer-term trend (Figure 10) (120). There is anecdotal evidence to suggest that, in some regions, inequalities in oral health are narrowing, however, particularly in areas where the COHS has a strong preventive approach.

However, caries prevention challenges persist. The ongoing consolidation of neoliberalism as the organizing principle of modern society has led to what has been coined the “neoliberal diet” (122): this is the energy-dense and nutritionally compromised industrial diet—highly processed and convenient “junk” food—which was the outcome of the U.S. agricultural subsidy policies of recent decades. Such food is high in sugar, salt, and fat. It has low nutritional value but is cheap, available, and requires minimal preparation. It tends to be consumed by those on low and/or insecure incomes, whose numbers are steadily rising as a consequence of neoliberal policies. Sugar intake is the most important dietary risk factor for dental caries (123), yet the marketing of sugar-laden food and drink continues unabated, with the true sugar content unapparent to most consumers. Attempts to restrict such marketing are strenuously resisted by food industry lobbying, despite the fact that some of the most popular supermarket products sold in New Zealand are less healthy (full-fat milk, white bread, sugary soft drinks, butter, and sweet biscuits) (124). Early childhood caries (ECC) shows no signs of disappearing and recent research shows that ECC is actually increasing. The numbers of children being treated under general anesthetic nationally have increased by 60% since 2004 and there are increases in ECC prevalence, for example, from 16.6% in 2009 to 23.3% in 2013 in Canterbury (125).

Addressing oral health inequalities, particularly in relation to Māori and Pacific Island children, was a priority for the new model of care. DHBs were encouraged to contract Māori and Pacific Health Providers to provide oral health services suited to the needs of their communities, for example, in the case of Māori, providing a whānau ora approach to care (119). This new vision/policy attempted to garner the participation of these communities in the design and implementation of the re-orientated COHS, identifying this as essential in addressing the social determinants of health. Although encouraged within GOHFAFL, in reality, financial constraints, historical and cultural institutionalism, and traditional oral health care delivery models reduced the full impact of this new policy in the most-affected communities. This has most likely resulted in a new service that is not innovative enough to overcome the structural determinants that exist in New Zealand society and which still excludes those who are most disadvantaged.

While the changes in service delivery and the effect on inequalities in oral health outcomes are yet to be fully examined, the Institute of Environmental Science and Research Limited (ESR) was contracted by the Ministry of Health in 2014 to evaluate and report on the reorientation of services. The Ministry of Health had set up a “Quality Improvement Group” for Māori Oral Health Providers and this group was interviewed as part of the evaluation (112). They felt that they had had variable input into the business plans, depending on which DHB they belonged to. They indicated that, while they had been consulted, the DHBs addressed their own needs, focusing on the aging workforce, equipment, and facilities, believing that this would address inequalities in oral health for Māori children. The new COHS, was “…just a retrofit of the old school dental system and a huge missed opportunity to do something really different,” such as implementing a whānau ora model where the whole whānau could be seen and treated together. Māori Oral Health Providers also had concerns about widening inequalities between Māori children and others, because Māori were more likely to live in rural areas and be socially disadvantaged and unable to travel to hub clinics (126). Dental therapists interviewed for this report, and another study conducted in the Southern region of New Zealand, also believed that those with the most need were less likely to be able to access clinical services due to unavailability of transport or parents being unable to take time off work to bring their children (126, 127).

## The Dental Therapy Workforce

There is evidence, however, to suggest that inequalities in oral health narrow during childhood and adolescence in New Zealand with access to free dental care and that these widen again after the age of 18 years (111, 115). Recent research, comparing adult oral health in several countries, has confirmed that indigenous people, including Māori, have a higher prevalence of decayed and missing teeth than their non-indigenous counterparts (128), and there are distinct social gradients in tooth loss (129). While the fact that oral health declines once New Zealanders must pay for their own dental care has been recognized for many years, previous Government policy has failed to address this issue. Both the New Zealand Health Strategy and GOHFAFL acknowledge that future policy work needs to focus on what care can be provided to low-income adults, with GOHFAFL suggesting that community-based facilities may develop the capability to provide services to lower-income adults (114, 119). While the 1988 Dental Act restricted dental therapists to working in public practice, there was no age limit on the patient group they could care for; dental therapists in some DHB areas treated low-income adults. Subsequent legislation (Health Practitioners Competence Assurance Act 2003) resulted in dental therapists being limited to caring for patients up to 18 years of age, with only a small number being eligible to register in an additional adult scope of practice. While New Zealand’s “oral health” graduates currently register as both dental hygienists and dental therapists, and can work in private and public practice, their restorative skills remain confined to patients aged under 18 years (130, 131). An opportunity existed to include these skills in the new “oral health therapy” scope of practice (to be implemented in November 2017); however, opposition at the consultation stage resulted in this being removed (132, 133).

The dental therapy workforce has been aging for some time, with the average age of the New Zealand dental therapist being over 50 years (117, 134). DHBs will need to further develop ways to recruit, and retain, dental and oral health therapists, particularly since New Zealand oral health therapists are also able to work in private practice and can practice both dental therapy and dental hygiene. Enabling graduates to use both sets of skills in a DHB setting will offer graduates a more attractive career path, and be of benefit to the patient group, particularly in caring for patients with special needs. GOHFAFL also advocates for a workforce representative of the ethnic diversity of the New Zealand population, as part of the effort to reduce inequalities. Numbers of Māori and Pacific students enrolled in the health professional degrees are increasing and they are being supported to study by innovative schemes such as the University of Otago’s Māori Health Workforce Development Unit’s “Te Whakapuāwai: Health Sciences First Year achievement programme” (135).

Dental therapists have been used to improve access to care for low-income adults and indigenous people elsewhere, for example, Alaska’s dental health aide therapists (dental therapists), the first of whom trained in New Zealand. In Australia, oral health therapists and dental therapists can treat up to the age of 25 years in their dental therapy scope. The 2009 New Zealand Oral Health Survey found that those of lower income, those aged 18–24 years, and Māori and Pacific Islanders were the least likely to attend for dental care with cost of care being a major factor (1). While there is no specific evidence that enabling oral health therapists to treat lower-income groups in the New Zealand context will improve access to care and make dental treatment more affordable or reduce inequalities, an opportunity to investigate this potential has been lost.

## Conclusion

The New Zealand SDS/COHS has proved itself very adaptable over the decades in its efforts to improve children’s oral health. Nevertheless, inequalities in oral health still exist for New Zealand children and adolescents, and these gaps widen in the adult years. The CSDH framework describes the health system as an intermediary determinant of health; it can directly affect health outcomes in its provision of access to care, and whether or not it promotes intersectoral action and social participation in decision-making, both of which are key to improving health and reducing inequalities. As such, the SDS (now the COHS) can also be considered a determinant of health, with SDS/COHS policy influencing both oral health status and oral health inequalities and, in turn, being shaped by a variety of social, political, and economic forces.

The CSDH framework notes that interventions to improve inequalities in health are often aimed at intermediary determinants of health. This has also been the case in terms of efforts by successive New Zealand Governments to reduce oral health inequalities. At the SDS/COHS level, policy has been directed at early intervention, increasing enrollment and access to care, as well as at increasing preventive care. These have had a positive effect on the oral health of children and adolescents in recent years; however, interventions aimed at the intermediary level often improve health indicators but leave health inequities unchanged. Efforts to reduce inequalities need to be directed at tackling the structural determinants of health, with policies paying attention to contextual specificities and using methodologies developed by social and political science.

Strategies to reduce inequalities must reach beyond the health sector in order to tackle the structural determinants of health. While some issues that affect health inequalities, such as poverty and poor living conditions, need intervention at a Government policy level, oral health services can drive policy change in other areas. In New Zealand, COHS and DHB leadership can create momentum for policy change by lobbying for a tax on sugar, dental treatment for low-income adults, and proposed legislative change to move the decision for fluoridating water supplies from local Government to DHBs.

The CSDH framework promotes social participation as being crucial to reducing inequity and empowering affected communities, and social participation is considered an ethical obligation in policy development. Disadvantaged communities need to not only be consulted and involved in policy decisions concerning their health but also be empowered to take control over these decisions. New Zealand’s Treaty of Waitangi requires that policymakers work together with Māori to develop strategies to reduce inequities in health that are relevant to Māori cultural concepts, values and practices. While some attempt has been made to involve Māori and Pacific Health Providers in policies affecting oral health, more effort is required to further reduce inequalities in oral health.

Ongoing monitoring of oral health and evaluation of oral health programs are essential in deciding what initiatives are likely to be most successful in reducing inequalities in the future. In the past, the failure to do so adequately has meant that SDS management was not always aware of oral health inequalities. In 2006, New Zealand’s strategic vision for oral health (GOHFAFL) identified “research, monitoring and evaluation” as one of several action areas but, more than a decade on, there is still much work to be done in this area. Finally, reviewing the history of a public service is a form of retrospective evaluation; it can help to avoid repeating mistakes of the past and aid in determining future policy. Furthermore, other countries can learn from New Zealand’s experience in their efforts to provide effective and accessible services to improve oral health and reduce oral health inequalities.

## Author Contributions

All authors, SM, LFP, and WT, have made both direct and intellectual contribution to the manuscript and all have approved it for publication.

## Conflict of Interest Statement

The authors declare that the research was conducted in the absence of any commercial or financial relationships that could be construed as a potential conflict of interest.

## Footnotes

^1^According to the Annual Reports of the Director-General of Health in the *Appendices to the Journals of the House of Representatives* H-31, 1931–1945.

^2^Pomare, being Māori, would use the word Pākehā to describe New Zealanders of European descent. The term varies depending on the publication. The authors have used the terms as stated in the original publications.

^3^The WHO goals were to be achieved by the year 2000. They were similar to the ones set at the Rotorua workshop (to be achieved by 1988) but the WHO had set a DMF of 3 for 12- to 13-year-olds, which New Zealand subsequently adopted.

^4^Hui Whakaoranga and Hui Tamata.

^5^Generations of New Zealand children referred to the school dental clinic as the “murder house,” due no doubt to the extent of treatment many required and the fact that, up until the late 1970s, local anesthetic was rarely used for filling work.
